# Automatic scar segmentation in dual inversion recovery images is more consistent with manual outlining than in conventional inversion recovery images

**DOI:** 10.1186/1532-429X-17-S1-O48

**Published:** 2015-02-03

**Authors:** Nicholas Byrne, Rene Botnar, Tarique Hussain, Stephen F Keevil, Sarah Peel

**Affiliations:** 1 Guy's and St. Thomas' NHS Foundation Trust, London, UK; 2 Division of Imaging Sciences and Biomedical Engineering, King's College London, London, UK

## Background

The dual inversion recovery (dual IR) pulse sequence has recently been shown to improve blood suppression and infarct delineation in late gadolinium enhancement (LGE) images of myocardial infarction. This resulted in significantly lower inter-observer variability in manual outlining of scar and higher expert confidence in scar detection and transmurality when compared with conventional inversion recovery (IR) images.

Computer algorithms have been shown to improve the accuracy of scar segmentation within IR images. We sought to develop and optimise a set of computer algorithms to quantify scar in both IR and dual IR images.

## Methods

**Figure 1:**
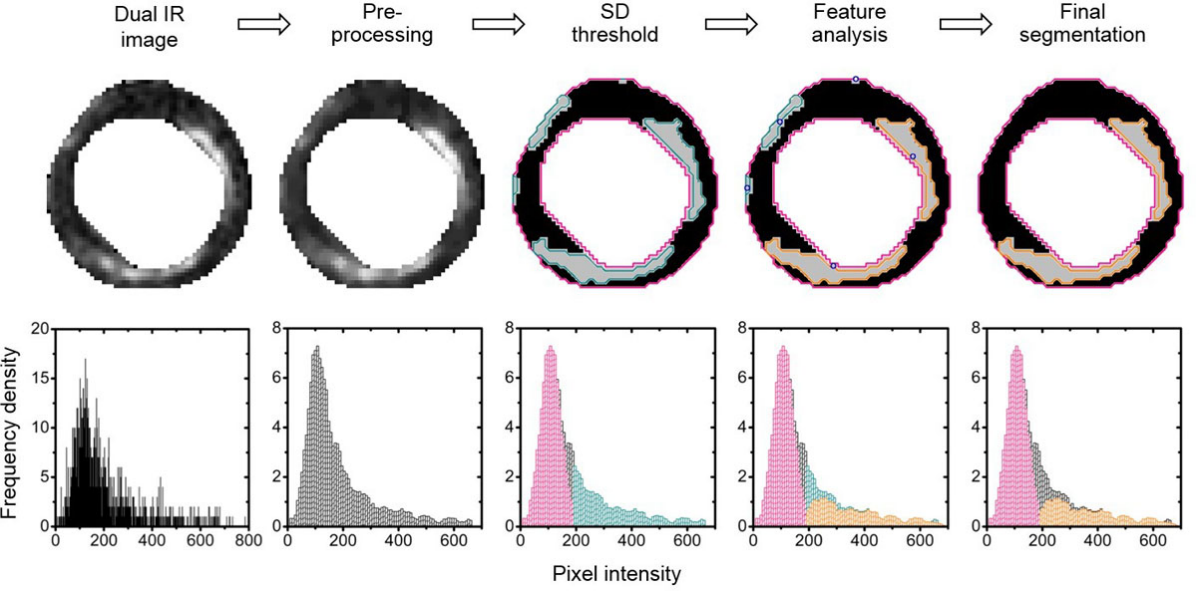
Reading from left to right, the steps used by the segmentation algorithm to separate scar from viable myocardium. The example shows the optimal segmentation of a dual IR DE-CMR image with a T1,min of 200 ms. Note that the myocardial cross-sections that form the input to this program have already been manually extracted from the original DE-CMR image. Under each step representative myocardial cross-sections and image histograms are shown. Features coloured in: pink, relate to the viable myocardium; green, to the pixels with intensity greater than the threshold; orange, to the pixels with intensity greater than the threshold and which belong to regions identified as scar by the feature analysis step. The blue rings indicate the weighted centroid for each aggregate of bright pixels that remains after standard deviation thresholding. These positions are used for subendocardial distance feature analysis.

A series of automated segmentation algorithms was developed for the separation of scar and healthy myocardium in IR and dual IR images. These rely on a combination of pre-processing, standard deviation thresholding and feature analysis (see Figure 1). Each stage has been optimised to match the manual segmentation of IR and dual IR images of 10 patients with chronic, left ventricular scar. Although the steps are not new in themselves, there are several novel aspects to the algorithms that have been developed. Importantly, the myocardial distribution of pixel intensities is computationally defined using a reflection method and does not rely on manual or region of interest input; this removes a source of subjectivity from pre-existing segmentation algorithms. Subendocardial distance feature analysis is implemented using a novel criterion that should perform equally well for ventricles of different sizes.

The Dice Similarity Coefficient (DSC) was used to assess agreement between algorithmic and manual segmentations, indicating optimal performance.

## Results

**Figure 2:**
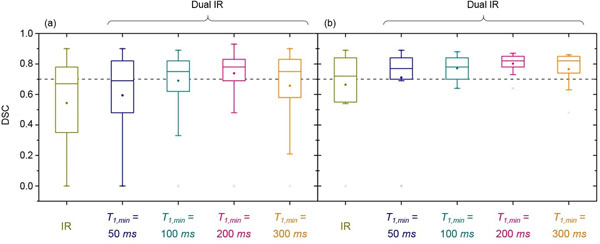
The effect of algorithm optimisation on images acquired using the different DE-CMR sequences. The DSC quantifies the similarity between manual and automated segmentation using: (a) all permutations of pre-processing and standard deviation threshold tested and (b) only values of these parameters chosen to maximise the agreement between the two segmentations. In each of the box plots the central line identifies the median, the dot gives the mean, the box gives the quartiles, the whiskers extend to the farthest data point within the upper and lower inner fence values and the crosses show the maximum and minimum results of the distributions. A DSC of more than 0.7 represents good agreement. Note the significant increase in agreement that is achieved by optimisation.

The mean DSC achieved by the Sequence Optimised Segmentation (SOS) algorithms exceeds 0.70, suggesting that the automated analysis of dual IR images is firstly possible and that excellent agreement between manual and automated segmentations is observed. For the dual IR sequence with *T_1_*_,_*_min_* = 200 *ms* the mean DSC is 0.80, higher than the value of 0.66 for the IR images (see Figure 2). The optimisation procedure suggests that in line with previous work, the pixel intensity threshold between normal myocardium and scar is between 2 and 3 standard deviations above the mean of the normal myocardium. Preliminary results for a subset of dual IR images also indicated that automated segmentation was possible without manual definition of the endocardial border and was sensitive to scar in the papillary muscles.

## Conclusions

The SOS algorithms quantify scar in both IR and dual IR images of the left ventricle. They show excellent agreement with manual segmentation performed by an expert cardiologist. Mean DSC for dual IR segmentation (0.80) exceeds that for IR images (0.66). This likely reflects the superior blood suppression of the dual IR sequence, allowing the expert to adopt a consistent approach to manual segmentation. Future work will focus on using the same methods to match algorithm performance to histology.

## Funding

The original study on which this work is based was funded by the British Heart Foundation Research Excellence Centre and the NIHR Biomedical Research Centre.

Please note that references have not been included due to concerns over institutional disclosure, but are available upon request.

